# Evaluation of metabolic response with ^18^F-FDG PET-CT in patients with advanced or recurrent thymic epithelial tumors

**DOI:** 10.1186/s40644-017-0112-x

**Published:** 2017-03-07

**Authors:** Sabrina Segreto, Rosa Fonti, Margaret Ottaviano, Sara Pellegrino, Leonardo Pace, Vincenzo Damiano, Giovannella Palmieri, Silvana Del Vecchio

**Affiliations:** 1 Department of Advanced Biomedical Sciences, University of Naples Federico II, Via Pansini 5, Edificio 10, 80131 Naples, Italy; 2 Institute of Biostructures and Bioimaging, National Research Council, Via T. De Amicis 95, 80145 Naples, Italy; 3 Rare Tumors Reference Center, University of Naples Federico II, Via S. Pansini 5, 80131 Naples, Italy; 4 Department of Medicine and Surgery, University of Salerno, Via S. Allende, 84081 Baronissi, Salerno Italy

**Keywords:** Thymoma, Thymic carcinoma, ^18^F-FDG PET-CT, Tumor response, RECIST

## Abstract

**Background:**

Patients with advanced or recurrent thymic epithelial tumors (TETs) often need several consecutive lines of chemotherapy. The aim of this retrospective monocentric study was to test whether ^18^F-Fluorodeoxyglucose positron emission tomography-computed tomography (^18^F-FDG PET-CT) is able to monitor standard chemotherapy efficacy in those patients and whether metabolic response correlates with morphovolumetric response as assessed by Response Evaluation Criteria in Solid Tumor (RECIST).

**Methods:**

We evaluated 27 consecutive patients with advanced (16 patients) or recurrent (11 patients) TETs. All patients underwent ^18^F-FDG PET-CT before and after at least 3 cycles of chemotherapy. Maximum standardized uptake value (SUV_max_) of all detected lesions was recorded and the most ^18^F-FDG avid lesion in each patient was selected for determination of percentage change of SUV_max_ (ΔSUV_max_) in pre- and post-treatment scans. Tumor response was assessed by contrast-enhanced computed tomography (CE-CT) using RECIST criteria. Receiver operating characteristic (ROC) curve analysis was performed to define the optimal threshold of ΔSUV_max_ discriminating responders from non-responders.

**Results:**

Metabolic response expressed as ΔSUV_max_ was significantly correlated with morphovolumetric response (Spearman’s rank correlation, *r* = 0.64, *p* = 0.001). ROC curve analysis showed that a ΔSUV_max_ value of -25% could discriminate responders from non-responders with a sensitivity of 88% and a specificity of 80%. Conversely, basal SUV_max_ values were not predictive of morphovolumetric tumor response.

**Conclusions:**

Our findings indicate that metabolic response assessed by ^18^F-FDG PET-CT, through evaluation of ΔSUV_max_, may allow identification of responders and non-responders thus guiding adaptation of therapy in patients with advanced or recurrent TETs.

## Background

Thymic epithelial tumors (TETs) are rare malignancies arising in the anterior mediastinum showing a high variable biological behaviour, from slow-growing benign lesions to highly aggressive carcinomas [1, 2]. According to the histological classification of the World Health Organization (WHO), TETs are subdivided into type A, AB, B1, B2, B3 and thymic carcinomas, characterized by an increasing degree of malignancy [1]. Thymic epithelial tumors are routinely staged according to Masaoka-Koga staging system, that considers the integrity of the thymic capsule (stage I), the micro or macroscopic invasion of surrounding tissues and organs (stage II and III), the presence of pleural or pericardial metastasis (stage IVA) and the lymphogenous or haematogenous metastatic spread (stage IVB) [3, 4]. Although both WHO classification and Masaoka-Koga staging system contribute to risk stratification of patients with TETs, therapeutic decisions are essentially taken on the basis of disease stage [5, 6] since WHO classification appeared to have a limited clinical predictive value [7].

The treatment strategy for thymic epithelial tumour is primarily based on whether the tumor can be radically resected or not at diagnosis [8–10]. Although surgery remains the treatment of choice, most of these tumors are unresectable or in advanced stages at diagnosis and require chemotherapy, eventually followed by surgery if tumors become resectable after the planned regimen. Furthermore, despite radical resection, recurrence is quite common in those patients and, although recurrent lesions are managed with the same approach used for newly diagnosed TETs, multicourse therapy is often necessary [8–10]. Cisplatin-based combination regimens are usually administered to patients candidate for both neoadjuvant and palliative chemotherapy [11–14]. Several consecutive lines of chemotherapy are also available for patients presenting tumor progression.

In this clinical context imaging modalities are of primary importance in the assessment of tumor resectability and for the evaluation of tumor response to chemotherapy. Contrast-enhanced computed tomography (CE-CT) is the routinely used imaging modality for diagnosis, staging and follow-up of TETs [15–19]. Furthermore in patients with advanced disease undergoing primary or definitive chemotherapy, CE-CT is usually performed to reassess resectability or to determine tumor response using Response Evaluation Criteria in Solid Tumor (RECIST) [20, 21].

Functional imaging with ^18^F-Fluorodeoxyglucose positron emission tomography (^18^F-FDG PET) with its ability to identify more aggressive and invasive subtypes of TETs provides useful information for the biologically characterization of thymic masses [22–25] and for disease stage [26–29]. Furthermore, ^18^F-FDG PET-CT has been performed to monitor the efficacy of targeted therapy in patients with advanced TETs and a reduction of ^18^F-FDG uptake higher than 30% closely correlated with objective tumor response [30]. Despite the wide use of ^18^F-FDG PET-CT in the assessment of metabolic response to standard chemotherapy in many solid tumors [31, 32], only few studies tested the ability of ^18^F-FDG PET-CT to identify responders and non-responders to standard chemotherapy in small series of patients with TETs [33–36]. Since metabolic response usually precedes the morphovolumetric reduction of tumor burden, the early detection of treatment failure may indicate the need to adopt alternative regimens [32, 37, 38]. The aim of the present study is to test whether ^18^F-FDG PET-CT performed in patients with advanced or recurrent TETs before and after standard chemotherapy may discriminate responders from non-responders and whether metabolic response correlates with morphovolumetric RECIST criteria of tumor response.

## Methods

### Patients and treatment

In this retrospective monocentric study we evaluated the medical records of twenty-seven consecutive patients, 18 male (mean age ± SD, 56 ± 12 y) and 9 female (mean age ± SD, 57 ± 11 y), with advanced (16 patients) or recurrent (11 patients) thymic epithelial tumors who had undergone whole-body ^18^F-FDG PET-CT before and after standard chemotherapy regimens. Histopathogical diagnosis was obtained in all patients and, based on WHO classification, 1 B1, 7 B2 and 7 B3 TETs along with 12 thymic carcinoma were included in the study. All patients were staged using the Masaoka-Koga staging system based on CE-CT findings at presentation: 3 patients had unresectable stage III, 5 were in stage IVA and 19 patients had stage IVB which included a high percentage of thymic carcinomas (11 patients).

Among the 27 patients, 16 had no chemotherapy before the basal ^18^F-FDG PET-CT whereas 5 patients received prior adjuvant or neoadjuvant chemotherapy and 6 were treated with definite chemotherapy for advanced, unresectable TET (Table 1). Furthermore, 5 patients underwent radiation therapy after surgery or in combination with chemotherapy. After the basal ^18^F-FDG PET-CT scan, platinum-based chemotherapy was administered to 23 patients (3-8 cycles, median 5); four additional patients with advanced disease who were in progression after platinum-based chemotherapy were treated with gemcitabine-capecitabine (at least 7 cycles).

**Table 1:** Clinical characteristics of patients (*N* = 27)

Characteristics	N_0_	%
Age (yr)		
Mean ± SD^*^ (range)	56 ± 11 (36–82)	
Gender		
Male	18	67
Female	9	33
Histopathology (WHO^a^ classification)		
B1	1	4
B2	7	26
B3	7	26
Thymic carcinoma	12	44
Stage at presentation (Masaoka-Koga)		
III	3	11
IV A	5	19
IV B	19	70
Platinum-based regimen		
Yes	23	85
No	4	15
Prior chemotherapy		
Prior therapy for advanced TETs^b^	6	22
Prior adjuvant/neoadjuvant therapy	5	19
No prior therapy	16	59
Prior surgical resection of primary tumor		
Yes	11	41
No	16	59
Prior radiotherapy		
Yes	5	19
No	22	81

^*^ *SD* Standard Deviation

^a^ *WHO* World Health Organization

^b^ *TETs* Thymic epithelial tumors

### Response evaluation

Contrast-enhanced CT scan of skull, neck, chest, abdomen and pelvis was performed at baseline and at the end of the planned regimen and the effects of chemotherapy were assessed using the RECIST version 1.1 [21]. Tumor response was defined as: complete response (CR) when there was disappearance of all lesions; partial response (PR) if there was ≥ 30% reduction in lesion size; progressive disease (PD) if there was increase of more than 20% in lesion size or appearance of a new lesion; stable disease (SD) when no PR and no PD occurred. For statistical purposes patients with CR and PR were grouped in the class of responders whereas patients with SD and PD were considered non-responders.

### ^18^F-FDG PET-CT Study

^18^F-FDG PET-CT scans were acquired after fasting for 8 h and 60 min after intravenous administration of ^18^F-FDG (350–370 MBq). The blood glucose level, measured just before tracer administration, was < 120 mg/dL in all patients. Dual-modality imaging was performed with a PET-CT Discovery LS scanner (GE Healthcare, Milan, Italy) consisting of a PET scanner and a four-row multidetector computed tomography (MDCT) system. MDCT scan was acquired using the following parameters: 4 × 5 mm collimation (140 kV, 80 mAs), 0.8 s rotation time, pitch of 1.5; when indicated, a fully diagnostic contrast-enhanced CT was performed. PET scan was subsequently performed in 2-dimensional mode using 4 min per bed position and six to eight bed positions per patient, depending on patient height. Iterative images reconstruction was completed with an ordered subsets-expectation maximization algorithm (2 iterations, 28 subsets). Attenuation corrected emission data were obtained using filtered back projection CT reconstructed images (Gaussian filter with 8 mm full width half maximum) to match the PET resolution. Transaxial, sagittal, and coronal images as well as coregistered images were examined using Xeleris software and then transferred in DICOM format to an OsiriX workstation (Pixmeo, Switzerland). All areas of focal ^18^F-FDG uptake visible on 2 contiguous PET slices at least and not corresponding to physiological tracer uptake were considered to be positive [39]. The SUV_max_ values of all lesions in the pre- and post-treatment scan were recorded by two board-certified nuclear medicine physicians and discrepancies between their assessments were resolved by consensus through discussion. The SUV_max_ value of the most metabolically active lesion in each examination was used to define the ΔSUV_max_ as follows: ΔSUV_max_ = [(SUV_max_ post – SUV_max_ pre)/SUV_max_ pre] × 100.

### Statistical analysis

Statistical analysis was performed using the software MedCalc for Windows, version 10.3.2.0, (MedCalc Software, Mariakerke, Belgium). Data are expressed as mean ± SD if not differently indicated. Unpaired Student’s *t* test was used to compare means of normally distributed data sets as assessed by Kolmogorov-Smirnov test. Spearman’s rank correlation was used to examine the association between ∆SUV_max_ and tumor response. Receiver operating characteristic (ROC) curve analysis was performed to estimate the best value of ∆SUV_max_ capable of discriminating responders from non-responders. A *p* value < 0.05 was considered statistically significant.

## Results

Patient characteristics are summarized in Table 1. Pre-treatment ^18^F-FDG PET-CT scan showed abnormal ^18^F-FDG uptake in all patients detecting a total of 77 lesions, including 18 mediastinal masses, 15 lymph nodes, 23 pleura/pericardial implants, 16 visceral lesions and 5 bone lesions, with an average of 2.85 ± 2.03 lesions per patient (range 1-8). The lesion with the highest SUV_max_ value in each patient was selected as the target lesion for the assessment of metabolic response; these 27 target lesions showed a mean size of 52.90 mm ± 21.24 mm. The SUV_max_ values of those lesions ranged between 3.3 (pleural implant) and 20 (thymic carcinoma) with a mean of 8.67 ± 4.89 (Table 2). Post-treatment ^18^F-FDG PET-CT showed reduction of FDG uptake in the target lesion of 19 patients and an increase of tracer accumulation in 8 patients (Table 2). None of the patients showing reduction of ^18^F-FDG in the target lesion showed the appearance of new site of abnormal ^18^F-FDG uptake whereas new metabolically active lesions (3 metastatic lymph nodes, 1 lung lesion, 3 pleural implants and 1 large vessel infiltration) were found in 5 out of 8 patients showing increased ^18^F-FDG accumulation in the target lesion.

**Table 2:** Pre- and post-treatment SUV_max_ and ΔSUV_max_ values compared to morphovolumetric tumor response assessed by RECIST

Patient	Target lesion	SUV_max_ pre^a^	SUV_max_ post^b^	ΔSUV_max_^c^ (%)	RECIST^d^
1	Mediastinal mass	10.00	7.50	−25	PR
2	Mediastinal mass	6.60	3.50	−47	PR
3	Mediastinal mass	13.00	6.20	−52	SD
4	Pleural implant	9.23	0.00	−100	CR
5	Mediastinal mass	4.10	3.00	−27	PR
6	Lung lesion	18.70	17.00	−9	SD
7	Lymph node	12.00	11.50	−4	SD
8	Pleural implant	5.80	2.10	−64	PR
9	Pleural implant	7.60	1.70	−78	PR
10	Lymph node	3.70	8.20	120	PD
11	Mediastinal mass	4.19	5.30	26	SD
12	Mediastinal mass	8.40	11.30	35	SD
13	Lung lesion	19.70	5.70	−71	PR
14	Mediastinal mass	4.20	4.60	10	PR
15	Mediastinal mass	5.00	3.00	−40	PR
16	Mediastinal mass	8.40	0.00	−100	CR
17	Pleural implant	4.00	4.80	20	PD
18	Mediastinal mass	15.60	18.5	19	PR
19	Mediastinal mass	7.70	2.20	−71	PR
20	Lung lesion	8.80	14.40	64	SD
21	Mediastinal mass	8.30	5.70	−31	PR
22	Lymph node	8.50	5.40	−36	PR
23	Pleural implant	3.30	1.90	−42	PR
24	Mediastinal mass	7.00	4.50	−36	SD
25	Mediastinal mass	5.60	1.80	−68	PR
26	Mediastinal mass	20.00	15.00	−25	PR
27	Mediastinal mass	4.80	6.70	40	SD

^a^SUV_max_ pre: pre-treatment maximum Standardized Uptake Value

^b^SUV_max_ post: post-treatment maximum Standardized Uptake Value

^c^ΔSUV_max_: percentage change in maximum Standardized Uptake Value

^d^ *RECIST* Response Evaluation Criteria in Solid Tumors, *CR* Complete response, *PR* Partial response, *SD* Stable disease, PD, Progressive disease

After treatment with standard chemotherapy, morphovolumetric tumor response was assessed by contrast-enhanced CT. Based on RECIST criteria, an objective tumor response was observed in 17 patients (2 CR and 15 PR) whereas in the remaining patients, 8 showed SD and 2 had PD (Table 2).

Normally distributed SUV_max_ values of pre- and post-treatment ^18^F-FDG PET-CT scan in responders and non-responders were expressed as mean ± SD and compared. SUV_max_ values of pre-treatment ^18^F-FDG PET-CT scan were not significantly different between responders and non-responders (8.80 ± 5.04 vs 8.45 ± 4.88, *p* = 0.8645). Conversely SUV_max_ values of post-treatment ^18^F-FDG PET-CT scan were significantly lower in responders as compared to non-responders (3.94 ± 3.62 vs 8.99 ± 4.34, *p* = 0.0038).

**Fig. 1:**
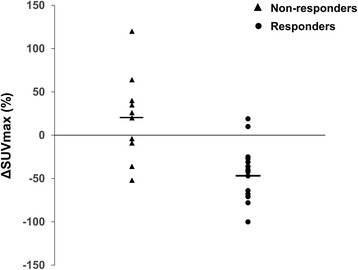
Distribution of ΔSUV_max_ values in patients allocated in the class of responders and non-responders by RECIST criteria. Responders showed ΔSUV_max_ values significantly lower than those of non-responders (*p* = 0.0003, unpaired *t*-test) and a significant correlation was found between ΔSUV_max_ values and morphovolumetric response (Spearman’s rank correlation, *r* = 0.64, *p* = 0.001). Horizontal bar indicates mean

The change of ^18^F-FDG uptake between baseline and post-treatment scan was -46.82% ± 8.10% (SE) in responders, indicating a reduction of tracer uptake, whereas non-responders showed an increase of ^18^F-FDG uptake with a ΔSUV_max_ of 20.40% ± 15.75% (SE). The normally distributed values of ΔSUV_max_ were significantly different in responders and non-responders (*p* = 0.0003, unpaired *t*-test) and were significantly correlated with morphovolumetric response (Spearman’s rank correlation, *r* = 0.64, *p* = 0.001). Fig. 1 shows the distribution of ΔSUV_max_ values in responders and non-responders.

**Fig. 2:**
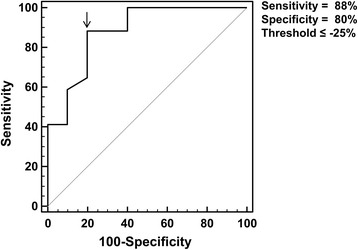
ROC curve analysis showed a sensitivity of 88% and a specificity of 80% in discriminating responders from non-responders (*arrow*) using a threshold of -25% for ΔSUV_max_

ROC curve analysis showed that a ΔSUV_max_ value of -25% could discriminate responders from non-responders with a sensitivity of 88% and a specificity of 80% (Fig. 2).

**Fig. 3:**
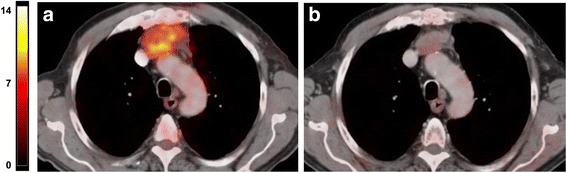
Representative images of baseline **a** and post-treatment **b** ^18^F-FDG PET-CT scan in a patient with thymic carcinoma. Fusion images of co-registered transaxial ^18^F-FDG PET and contrast-enhanced CT sections are shown. In the baseline scan SUV_max_ was 6.60 whereas the post-treatment study showed a SUV_max_ of 3.50. A 47% reduction of ^18^F-FDG uptake was found in this patient with partial response based on RECIST. The same maximum threshold of SUV was applied to PET images from pre-treatment and post-treatment scans as shown by the color scale on the left

**Fig. 4:**
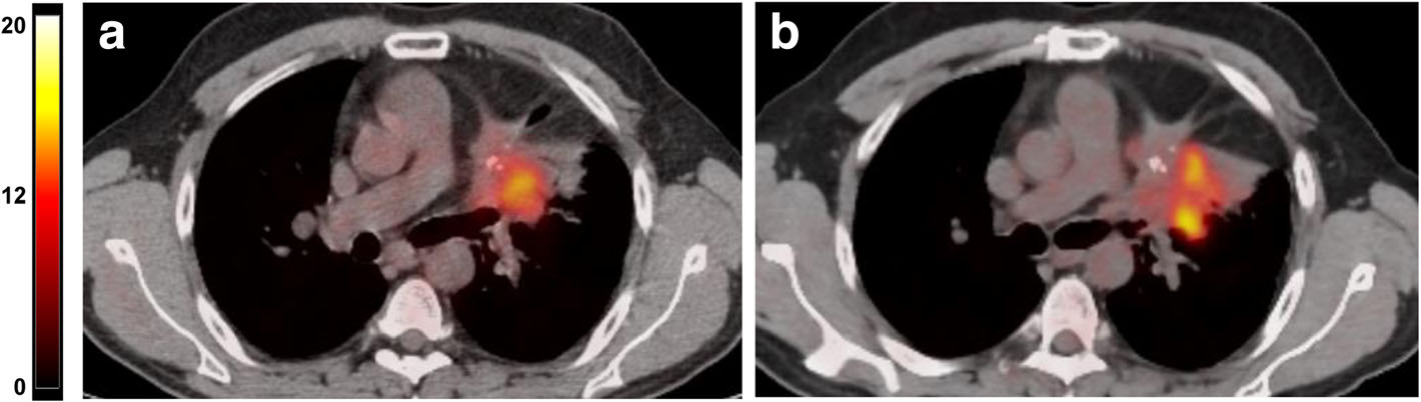
Representative images of baseline **a** and post-treatment **b** ^18^F-FDG PET-CT scan in a patient with thymic carcinoma. Fusion images of co-registered transaxial ^18^F-FDG PET and CT sections are shown. In the baseline scan SUV_max_ was 8.80 whereas the post-treatment study showed a SUV_max_ of 14.40. A 64% increase of ^18^F-FDG uptake was found in this patient with stable disease based on RECIST. The same maximum threshold of SUV was applied to PET images from pre-treatment and post-treatment scans as shown by the color scale on the left

Figures 3 and 4 show representative ^18^F-FDG PET-CT images of baseline and post-treatment scans in a patient with metabolic response and a patient with metabolic progression of the disease, respectively. The responding patient of Fig. 3 was allocated in the class of PR using RECIST and showed a 47% reduction of tracer uptake indicating a concordance between morphologic and metabolic tumor response. Conversely the non-responding patient of Fig. 4 was judged to have stable disease by RECIST but he showed a 64% increase of ^18^F-FDG uptake indicating a metabolic progression.

## Discussion

The present study showed that ^18^F-FDG PET-CT may be used to monitor tumor response to standard chemotherapy in patients with advanced or recurrent TETs. The percentage change of ^18^F-FDG uptake between baseline and post-treatment scans was indeed able to discriminate responders from non-responders and significantly correlated with tumor response assessed by RECIST criteria. In particular, a 25% reduction of ^18^F-FDG uptake identified responders with a sensitivity of 88% and a specificity of 80%. Our findings are in agreement with previous studies evaluating early metabolic response in patients with TETs mainly treated with targeted therapy [30]. All patients in our study received conventional chemotherapy for advanced or recurrent disease and, being potentially candidate to several consecutive lines of chemotherapy, tumor response was carefully assessed to guide subsequent therapeutic options.

Assessment of tumor response in patients with TETs is usually performed using RECIST criteria in which unidimensional tumor measurements are obtained from pre-and post-treatment CT scans to evaluate changes of tumor burden in response to therapy [20, 21]. Although RECIST criteria are widely accepted as the standard method to evaluate tumor response in solid tumors, they have some limitations in TETs. In fact, TETs differ from other solid tumors in terms of growth and dissemination patterns especially in advanced stages. They are often large masses with indefinite borders encasing mediastinal structures and infiltrating adjacent tissues. Furthermore non-contiguous pleural metastases are common in these patients and measurements of these lenticular lesions may be difficult. In order to overcome these limitations, International Thymic Malignancy Interest Group proposed modified RECIST criteria for the assessment of tumor response in TETs taking into account the peculiar growth and dissemination patterns of the disease [40–44].

Although standard criteria for the assessment of objective tumor response remain based on anatomical measurements, functional imaging with ^18^F-FDG-PET-CT has been used for the evaluation of metabolic response to therapy in many solid tumors. Previous studies showed indeed that conventional cytotoxic agents, by inducing tumor cell death, cause a reduction of cell viability and glucose demand with a consequent decrease of ^18^F-FDG uptake that may precede tumor shrinkage as assessed by anatomical measurements [32, 37, 38, 45, 46]. Due to the consistent results of a number of studies, recommendations on the use of ^18^F-FDG-PET for monitoring efficacy of therapy have been published and include EORTC (European Organization for Research and Treatment of Cancer) and PERCIST (PET Response Criteria in Solid Tumors) criteria which are based on changes of tracer uptake in response to treatment [31, 47]. Although clinically relevant thresholds have been proposed to classify metabolic response [48, 49] the optimal cut-off to discriminate responders and non-responders may vary among different malignancies depending on their tracer uptake patterns and dynamics during therapy. In our study, the optimal threshold that identifies responding and non-responding TETs is in agreement with the values proposed by both EORTC and PERCIST recommendations.

Despite the large use of ^18^F-FDG in the evaluation of metabolic response of solid tumors to therapy, it is still not clear how many lesions should be included in the analysis of pre and post-treatment PET scans especially in advanced stages. Previous studies reported analysis of both single and multiple lesions and both approaches resulted to be predictive of morphovolumetric response or outcome [48, 50]. Considering the potential association between ^18^F-FDG uptake and degree of invasiveness of TETs and the possible coexistence of different WHO histotypes in the same tumor mass, we decide to analyse the most ^18^F-FDG avid lesion in pre and post-treatment scans in order to derive the percentage change of tracer uptake, after ensuring that no new lesions were found or metabolic progression occurred in all other lesions in post-treatment ^18^F-FDG PET-CT. This simplified approach may be easily employed to evaluate metabolic response in patients with TETs in daily clinical practice although we are aware that other volume-based metabolic parameters, such as metabolic tumor volume (MTV) and total lesion glycolysis (TLG) may better reflect metabolic response in all lesions and be more reliable predictive markers of survival [51].

Limitations of our monocentric study are the retrospective analysis of imaging findings and the relatively limited series of patients. Therefore further studies are needed to confirm our findings in a larger population of patients and, since TETs are rare tumors, this may require the involvement of several institutions.

## Conclusions

Our study showed that metabolic response assessed by ^18^F-FDG PET-CT may complement RECIST criteria in the identification of responders and non-responders thus providing an additional guide for adaptation of therapy in patients with advanced or recurrent thymic epithelial tumors.

## Abbreviations

^18^F-FDG PET-CT: ^18^F-Fluorodeoxyglucose Positron Emission Tomography-Computed Tomography
CE-CT: Contrast-Enhanced Computed Tomography
CR: Complete Response
EORTC: European Organization for Research and Treatment of Cancer
MDCT: Multidetector Computed Tomography
MTV: Metabolic Tumor Volume
PD: Progressive Disease
PERCIST: PET Response Criteria in Solid Tumors
PR: Partial Response
RECIST: Response Evaluation Criteria in Solid Tumor
ROC: Receiver Operating Characteristic
SD: Stable Disease
SUV_max_: Maximum Standardized Uptake Value
TETs: Thymic Epithelial Tumors
TLG: Total Lesion Glycolysis
WHO: World Health Organization
ΔSUV_max_: percentage change of SUV_max_ in pre- and post-treatment scans.
